# A Theoretical Comparative Study of Vapor-Compression Refrigeration Cycle using Al_2_O_3_ Nanoparticle with Low-GWP Refrigerants

**DOI:** 10.3390/e24121820

**Published:** 2022-12-13

**Authors:** Shengyu Li, Jun Lu

**Affiliations:** School of Civil Engineering, Chongqing University, Chongqing 400045, China

**Keywords:** vapor-compression refrigeration cycle, COP, exergy efficiency, nanoparticles, nanorefrigerants

## Abstract

Nanorefrigerant is a mixture of nanoparticles and pure refrigerant, which can increase heat transfer characteristics in refrigeration and air conditioning equipment. The performance of four different Al_2_O_3_ nanorefrigerants and their pure fluids (R600a, R134a, R1234yf, and R1233zd(E)) is analyzed in a vapor-compression refrigeration cycle. The enthalpy of a nanorefrigerant in the refrigeration cycle is calculated by using the prediction method based on the density of nanorefrigerant. A numerical model is established for the thermodynamic analysis, and the results show that adding nanoparticles to the pure refrigerant enhances heat transfer in heat exchangers, increases cooling capacity, reduces compressor power consumption, and finally improves the performance of the refrigeration system. The COP improvement of R1233zd(E) + Al_2_O_3_ nanorefrigerant is the highest, and the COP improvement of R134a + Al_2_O_3_ and R1234yf + Al_2_O_3_ are close to each other. When the mass fraction of Al_2_O_3_ nanoparticles increases to 0.30%, the COP of R1233zd(E) and R600a increases by more than 20%; the maximum exergy efficiency is 38.46% for R1233zd(E) + Al_2_O_3_, and the minimum exergy efficiency is 27.06% for pure R1234yf. The results provide a basis for the application of nanorefrigerants in the vapor compression refrigeration cycle.

## 1. Introduction

One of the most important utilities for people’s daily lives is refrigeration systems. The vapor-compression refrigeration cycle (VCRC) is widely used in domestic and industrial sectors due to its higher Coefficient of Performance (COP) [1,2,3]. VCRCs have a share of about 30% of the total world energy consumption, and this ratio may increase due to refrigerant leakage [4,5]. The performance of the refrigeration system can be improved either by increasing the rate of heat absorption in the evaporator or by reducing the compressor power.

In 1995, Choi of the Argonne National Laboratory observed that mixing nanoparticles in a base fluid produces a thermally enhanced fluid called a nanofluid [6]. Since then, nanofluids have received the attention of many researchers around the world. Research on the enhanced heat transfer capacity provided by some nanoparticles dispersed in the base fluid has attracted interest among nanotechnology researchers. Nanoparticles can be synthesized by many methods, including chemical, physical, and biological approaches [7]. The use of metallic and semiconductor nanoparticles has been proven to enhance the heat transfer properties since the surface area and specific heat increase. In summary, for the nanorefrigerants, the specific heat increases with the temperature and decreases with the concentration; the thermal conductivity increases with the increase of concentration and temperature; the viscosity and density increase with the augmentation of concentration and decrease with the increment of temperature [8,9,10]. For more information on the preparation methods (single-step and two-step), thermophysical properties, heat transport mechanism, tribological behavior and stabilities of these nanorefrigerants, please refer to review publications of Table 1. 

The performance of the refrigeration system is directly related to the thermophysical properties of the refrigerant. Improving the thermophysical properties of the refrigerant can improve the system’s performance. Besides, the performance of VCRCs can be improved by ejector application, suitable refrigerant selection, operating conditions optimization, recovering the waste heat, and cycle configuration [16,17,18]. 

The emphasis of this study is to analyze the impact of the use of nanorefrigerants on the overall system performance. Studies on the performance of nanorefrigerants in VCRC are presented in Table 2. It is found that the most preferred nanoparticle is Al_2_O_3,_ and it is followed by TiO_2_, SiO_2,_ and CuO nanoparticles because of their stability characteristics in the base refrigerant; the most preferred refrigerant is R134a, followed by R600a.

Furthermore, Bellos and Tzivanidis [29] studied an absorption refrigeration system operating with the LiBr-H_2_O working pair driven by a solar collector. Pure water and water + Cu nanofluid (2 vol.%) are the examined working fluids on the solar field. The results showed that the mean thermal efficiency has an increase of nearly 2.5%. The daily exergetic performance and refrigeration production is increased to 4.0% and 0.84%, respectively, with the use of nanofluids in the solar collector. Hamrahi et al. [30] studied the influence of nanoactivated carbon on the property of solar adsorption chillers with two beds adsorption refrigerators. Adding nanoactivated carbon with concentrations of 4.7 wt.%, 11.1 wt.%, and 18.3 wt.% to the adsorption bed under 30 °C and 34 °C can increase COP by 11% and 21%, 33% and 17%, 23%, and 25%, respectively. Tashtoush et al. [31] studied the effect of nanoparticles on the COP of the ejector refrigeration cycle. The results showed that the improvement in COP reaches 24.7% and 12.61% for R134a with 2 wt.% CuO and Al_2_O_3_, respectively, while the refrigerant vapor mass at evaporator exit increases from 0.7616 for pure R134a to 0.8212 for R134a + 0.2 wt.% CuO nanorefrigerant. Moreover, Azmi et al. [32] reviewed the impact of nanorefrigerant and nanolubricant on energy saving in refrigeration systems. Sharif et al. [33] reviewed the mechanism for improvement in VCRC performance with nanorefrigerants and nanolubricants. Bhattad et al. [34] summarized the applications of nanofluids as refrigerants, lubricants, and secondary fluids in refrigeration systems. Jiang et al. [35] discussed the nanofluid advantages in absorption refrigeration systems. 

Although a large number of numerical and experimental studies on nanofluids have been conducted, most of the investigations focus on the fundamental properties and heat transfer characteristics of nanorefrigerants. There are limited studies on the evaluation of the refrigeration cycle efficiency with nanorefrigerants, especially few reports on the applicability of a nanoparticle-enhanced vapor-compression refrigeration cycle. Also, nanorefrigerant studies are typically experimental due to the absence of nanorefrigerant state equations. In the present analysis, the feasibility of four refrigerants, R600a, R134a, R1234yf, and R1233zd(E), containing Al_2_O_3_ nanoparticles for the vapor-compression refrigeration cycle is carried out numerically. With a mass fraction of 0.1% Al_2_O_3_ nanoparticles, the performance of these four nanorefrigerants is evaluated for various evaporation and condensation temperatures. The system performance is mainly presented in terms of COP and exergy efficiency. In addition, the effect of different mass fractions of nanoparticles is investigated. It provides a reference for the application of nanorefrigerant in refrigeration equipment such as domestic refrigerators and air conditioners. 

## 2. Methodology

**Figure 1:**
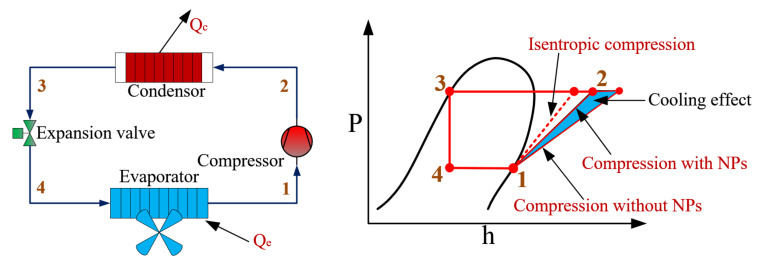
Schematic and P-h diagram of the refrigeration cycle.

The principles of a refrigeration cycle are no different from standard VCRC. The simulation is mainly to calculate the effect of nanoparticles (NPs) on the refrigerant’s physical parameters and to correct the compression process model. The refrigeration system schematic and the corresponding P-h diagram are shown in Figure 1. In this study, the nanorefrigerant model is based on the work of Aktemur et al. [36], Aktas et al. [37], and Javadi [28], and several assumptions from the work of Tashtoush et al. [31] and Bellos et al. [29] are made to simplify the model: (1) Heat loss and pressure drop of the working fluid during heat transfer are not considered. (2) The working fluid is saturated at the evaporator and the condenser outlet. (3) The NPs are uniformly distributed in the gas and liquid phases of the working fluid without aggregation and sedimentation effects. (4) The refrigerant and NPs are at the same temperature in each component. 

### 2.1. Nanorefrigerant Model 

The properties of the four preliminary selected refrigerants are shown in Table 3. International protocols such as Kyoto (1997) and Montreal (1987) restrict the usage of chlorofluorocarbons (CFCs) and hydrochlorofluorocarbons (HCFCs) in VCRCs despite their lower price. Most developed countries ruled out the options of CFCs in refrigeration. While HCFCs are considered for short-term (transitional) use, hydrofluorocarbons (HFCs) are preferred for long-term applications. HCFC refrigerants are expected to be phased out in developing countries (by 2040) and developed countries (by 2030). HFCs are phased down due to the F-Gas regulation. As a result, CFCs, HCFCs, and HFCs will be phased out in the near future due to their adverse effects on the environment. After 2010, the fourth generation of refrigerants (hydrofluoroolefins HFOs) was introduced with the main purpose of focusing on low GWP, low ODP, and short lifetime [38]. In addition to HFOs, hydrochlorofluorocarbon olefins (HCFOs) are also thought to have low ODP and extremely short life spans, as well as very low GWP. From an environmental and economic sustainability perspective, hydrocarbons (HCs) are considered to be the best choice of refrigerant. 

The HFC-134a currently remains the most commonly used refrigerant in vehicle air conditioning, large air/water cooling units, or high-temperature heat pump systems because of its excellent thermal-physical properties and low cost. After 2022, R134a will be banned in automotive air conditioners because the GWP value is as high as 1430. Eco-friendly refrigerants, such as R1234yf, R1234ze, and R152a, have the possibility of directly replacing R134a due to similar thermodynamic properties [42]. The HC-290 and HC-600a can be used as alternatives to the R134a in household refrigerators. HFO-1234yf is the best alternative to R134a in automobile air conditioning systems. R-1233zd(E) is expected to replace R134a as a low-temperature heat pump cycle refrigerant due to its similar physical properties. 

Nanorefrigerants (NRs) can be prepared by adding carbonaceous material, metal, metal oxides, conducting polymers, nanocomposite, or hybrid nanoparticles into the pure refrigerant. The thermophysical properties of the NR depend on the thermophysical properties of the pure refrigerant and NPs, such as density, specific heat, thermal conductivity, viscosity, mixture ratio, and concentration. The percentage of the most preferred NPs is summarized in Table 4 [43]. The preferred NP is by far Al_2_O_3,_ with 36.84% of research work; other commonly used NPs include CuO, TiO_2,_ and SiO_2_. The thermal conductivity, density, and specific heat of NPs are not dependent on their size. As shown in Table 5, volume fraction (φ) is commonly used to calculate the thermophysical properties of NPs. Due to the difficulty of measuring the exact volume of NPs, the volume fraction (φ) can be corrected by the mass fraction (ω). 

The density of the NR can be used as a metric to calculate the enthalpy because there are no characteristic correlations of the NRs. The parameter n in the thermal conductivity expression ($k_{NR}$) depends on the shape of the particles. The density ($\rho_{NR}$) of the NR is higher than that of the single refrigerant, while the specific heat capacity ($c_{p,\;NR}$) is lower. In addition, effective dynamic ($\mu_{NR}$) and thermal conductivity ($k_{NR}$) are slightly increased but not constant at different components [47]. 

### 2.2. Energy Model

The general energy balance equation can be defined as:

(1) $$Q_{in}-\sum W_{out}+\sum m_{in}h_{in}-\sum m_{out}h_{out}=0$$

Based on the first law of thermodynamics, the thermodynamic equations of each component are obtained, as shown in Table 6. The compressor isentropic efficiency ($\eta_{is}$) adopts the pressure ratio ($P_{2}/P_{1}$) correlation formula, which obtains the exhaust temperature and the compressor power consumption ($W_{comp}$). The NPs absorb heat from the refrigerant during the compression process, with the aim of achieving approximately isentropic compression. This effect is similar to the injection of oil or water in the compressor cylinder, which can reduce the discharge temperature and improve the compressor’s performance. 

### 2.3. Exergy Model

When the kinetic and potential energy changes are neglected, the exergy at each state point can be defined as:

(2) $$Ex=m\left[\left(h-T_{0}s\right)-\left(h_{0}-T_{0}s_{0}\right)\right]$$

The general exergy balance can be expressed as:

(3) $$Ex_{des}=Ex_{in}-Ex_{out}+\sum {\left[Q\left(1-\frac{T_{0}}{T}\right)\right]}_{in}-\sum {\left[Q\left(1-\frac{T_{0}}{T}\right)\right]}_{out}+\sum W_{in}-\sum W_{out}$$

Based on the second law of thermodynamics, the exergy destruction ($Ex_{des}$) and exergy efficiency ($\eta_{ex}$) of each component is listed in Table 7. The dead state of the working fluid is at pressure $P_{0}=100\;\mathrm{kPa}$ and temperature $T_{0}=30\;{}^{\circ}C$. 

### 2.4. Performance Evaluation 

The *COP* for the system is calculated by:

(4) $$COP=\frac{Q_{eva}}{W_{comp}}$$

The total exergy destruction is expressed by:

(5) $$Ex_{dest,\;tot}=Ex_{des,\;comp}+Ex_{des,\;con}+Ex_{des,\;exp}+Ex_{des,\;eva}$$

Also, the exergy efficiency can be described by:

(6) $$\eta_{ex}=\frac{Ex_{out}}{Ex_{in}+W_{comp}}=1-\frac{Ex_{dest,\;tot}}{W_{comp}}$$

Relative irreversibility (*RI*) can be defined for the component:

(7) $$RI=\frac{Ex_{des,\;component}\;}{Ex_{dest,\;tot}}$$

In addition, improvements in the COP and $\eta_{ex}$ of the nanorefrigerant system over the pure refrigerant system are evaluated:

(8) $$COP_{imp}=\frac{COP_{NR}-COP_{R}}{COP_{R}}$$

(9) $$\eta_{ex,\;imp}=\frac{\eta_{ex,\;NR}-\eta_{ex,\;R}}{\eta_{ex,\;R}}$$

## 3. Model Validation

To verify the accuracy of the proposed model, the COP for the pure R134a and R134a + SiO_2_ nanorefrigerants are calculated with the same operating conditions in [50] (evaporation and condensation temperatures of −7 °C and 42 °C, respectively). It should be noted that the $h_{1,NR}$ is calculated as follows: Firstly, evaporation temperature ($T_{1,R}$) and saturation pressure ($P_{1,R}$) are set to −7 °C and 225 kPa, respectively. Then, the density of pure refrigerant ($\rho_{1,R}$) is calculated from the CoolProp database. Based on the density of $\rho_{1,NP}$ and the $\rho_{1,R}$ and the mixture ratio, the density of nanorefrigerant ($\rho_{1,NR}$) can be estimated. Last, the enthalpy, entropy, temperature, and pressure can be determined for point 1. The condensation temperature ($T_{3,R}$) and saturation pressure ($P_{3,R}$) are specified as 42 °C and 1072 kPa, respectively, for point 3, and the thermophysical parameters at point 3 are determined in similar methods as point 1. The $h_{4,NR}$ at the evaporator inlet is equal to $h_{3,NR}$ due to adiabatic expansion in the expansion valve. The thermophysical parameters at point 2 are determined by $h_{1,NR}$ and $s_{1,NR}$. The $h_{1,NR}$ and $h_{3,NR}$ are summarized in Table 8. It can be found that the simulated COP and Akhayere et al. [51] data show the same trend: an increase in the COP with the SiO_2_ nanoparticles. A rise of 23.46% in COP is obtained by Hussin et al. [50], while an increase of 20.32% is recorded in this study. The deviation of the theoretical COP is not greater than ±5%, and the average deviation is −1.21%. Although small deviations exist, it still proves that the model works well enough to make accurate predictions. Moreover, according to the conclusion of Hussin et al. [50], by increasing NP mass fraction up to 0.5%, the COP value is increased and later decreased as compared to the pure R134a. Increasing the amount of NPs can improve the heat transfer performance of the cooling system. A high number of NPs increases the pressure drop of the refrigerant and correspondingly increases the required pumping power.

## 4. Results and Discussion

### 4.1. Comparative Study under Typical Operating Conditions

Table 9 provides related properties and results for the cycle working with four selected pure refrigerants and NRs under typical operating conditions of $T_{1}=0\;{}^{\circ}C$, $T_{3}=45\;{}^{\circ}C$ (the mass fraction of the NP is 0.1%). Thermophysical properties like density, pressure, enthalpy, and entropy of refrigerants can be predicted by general form-state equations. The enthalpy of NR at point 1 is slightly higher than that of pure refrigerant. Enthalpy at point 3 for the NR is slightly below that of pure refrigerant. Enthalpy and temperature at point 2 are slightly lower than those in the pure refrigerant system, mainly attributed to the cooling effect of the Al_2_O_3_ NPs during the compression process. The obtained NR density is slightly increased compared to the pure refrigerant density. It is also discovered that adding Al_2_O_3_ NPs to the pure refrigerants enhances the evaporator heat per unit mass ($q_{e}$) and evaporator heat per unit volume ($q_{v}$). The $q_{e}$ of R600a is observed to be the highest, and the lowest values are obtained in the case of R1234yf. The $q_{v}$ of R1233zd(E) is the smallest, while R134a is the highest. A high $q_{v}$ indicates that a small compressor is required, reducing the initial investment costs of the refrigeration system. The discharge temperature of R134a is higher than that of R600a, R1234yf, and R1233zd(E). A lower discharge temperature increases the longevity of the compressor and reduces oil aging. R134a has the lowest compressor efficiency, while R1233zd(E) has the highest compressor efficiency. 

Compared with the pure refrigerants R600a, R134a, R1234yf, and R1233zd(E), the compressor work per unit mass ($w_{comp}$) with 0.1% Al_2_O_3_ nanoparticles is decreased by 2.96%, 2.80%, 2.89%, and 6.13%, respectively; and the corresponding COP is increased by 6.36%, 3.10%, 3.32%, and 6.92%, respectively. The COP of R1233zd(E) decreases to a larger extent than the other refrigerant. An increase in COP is caused by an increase in $q_{e}$ and a decrease in $w_{comp}$. The COP values of R600a and R1233zd(E) are greater than that of R134a, while the COP value of R1234yf is smaller than that of R134a. Among the NRs studied, the R1233zd(E) + Al_2_O_3_ has the highest COP value, while the R1234yf + Al_2_O_3_ has the lowest COP value. In the case of R134a + Al_2_O_3_, the COP is 11.41% less than that of R1233zd(E) + Al_2_O_3_, it is 5.41% lower than that of R600a + Al_2_O_3_, and it is 0.60% higher than that of R1234yf + Al_2_O_3_. The R1233zd(E) with Al_2_O_3_ nanoparticles may be used as a replacement for R134a because of zero ODP, low GWP, and the most appropriate thermodynamic characteristics. 

### 4.2. Performance Analysis under Variable Operating Conditions

It should be noted that the problem of maximizing cycle performance and the constraints can be described as follows: The evaporation temperature ranges from −10 °C to 20 °C, the condensation temperature varies from 25 °C to 55 °C, the mass fraction of Al_2_O_3_ nanoparticles ranges from 0 to 0.3%.

#### 4.2.1. Evaporator Temperature

**Figure 2:**
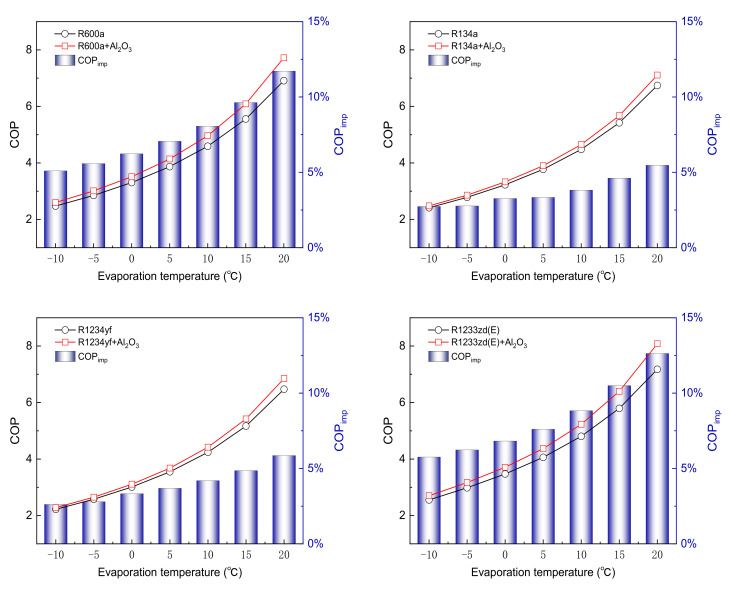
Variation of COP with evaporation temperature at condensation temperature of 45 °C.

Figure 2 shows the effects of evaporation temperature ($T_{e}$) on the COP at 45 °C condensation temperature ($T_{c}$). With $T_{e}$ varying from −10 °C to 20 °C, the COP for the pure refrigerants R600a, R134a, R1234yf, and R1233zd(E) increases from 2.47 to 6.91, 2.41 to 6.74, 2.22 to 6.47, and 2.55 to 7.17, respectively; the incremental values of COP are approximately 0.148, 0.144, 0.142, and 0.154 ${{}^{\circ}C}^{-1}$, respectively. For the NR system, the corresponding COP increases from 2.6 to 7.72, 2.48 to 7.11, 2.28 to 6.85, and 2.7 to 8.08, respectively; the COP increase by 0.171, 0.154, 0.152, and 0.179 for every 1 °C increase in $T_{e}$, respectively. It can be found that the rate of COP increment for the NR system is more sensitive to $T_{e}$ than that of a pure refrigerant system. The lowest increase in the COP is observed for R1234yf, and the largest increase is found for R1233zd(E). The rise in evaporator temperature decreases the pressure ratio, and hence less compressor work input. The trend of COP remains the same as NPs added to the pure refrigerant. In addition, the minimum value of COP is found for R1234yf and R1234yf + Al_2_O_3_ at $T_{e}$ of −10 °C, and the maximum value is found when $T_{e}$ is 20 °C for R1233zd(E) and R1233zd(E) + Al_2_O_3_. It shows that the use of NP does not change the inherent characteristics of the pure refrigerant. Furthermore, the improvement ratio of COP increases with the $T_{e}$. Compared with the pure refrigerants R600a, R134a, R1234yf, and R1233zd(E), the ${\mathrm{COP}}_{\mathrm{imp}}$ for the NPs is increased by 5.09–11.72%, 2.73–5.47%, 2.61–5.86%, and 5.76–12.63%, respectively; the minimum value of ${\mathrm{COP}}_{\mathrm{imp}}$ is observed when $T_{e}$ is −10 °C for R1234yf, and the maximum value of ${\mathrm{COP}}_{\mathrm{imp}}$ is found when $T_{e}$ is 20 °C for R1233zd(E). The increase is lowest for R134a and highest for R1233zd(E) at various evaporation temperatures.

**Figure 3:**
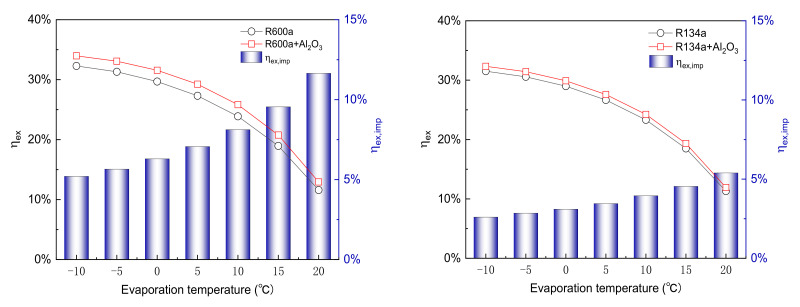

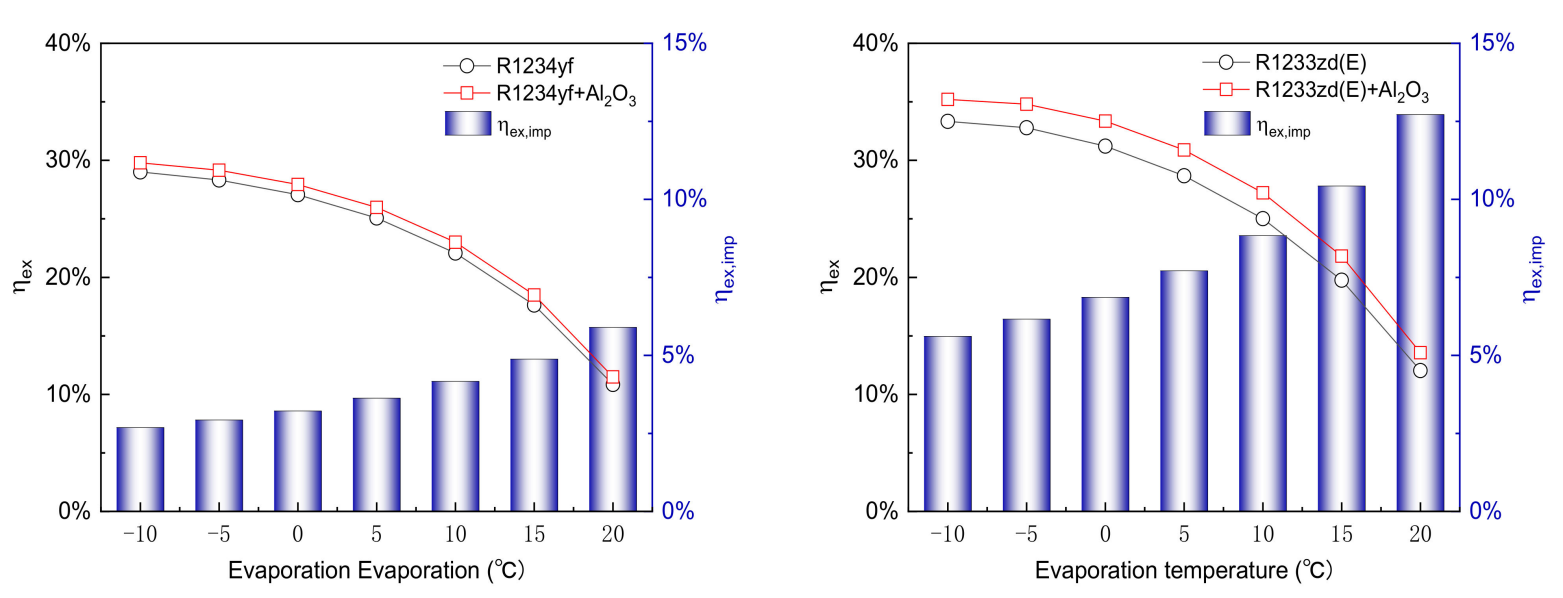
Variation of exergy efficiency with evaporation temperature at condensation temperature of 45 °C.

Figure 3 shows the variation in exergy efficiency ($\eta_{ex}$) with $T_{e}$ varying from −10 °C to 20 °C at $T_{c}$ of 45 °C. The range of changes in $\eta_{ex}$ for the pure refrigerants R600a, R134a, R1234yf, and R1233zd(E) is 32.29% to 11.59%, 31.51% to 11.31%, 29.01% to 10.85%, and 33.33% to 12.03%, respectively. For the NR system, the maximum values of $\eta_{ex}$ are 33.97%, 32.33%, 29.79%, and 35.20%, respectively; the corresponding minimum values of $\eta_{ex}$ are 12.94%, 11.92%, 11.49%, and 13.56%, respectively. It can be seen that the $\eta_{ex}$ of the NR system is higher than that of the pure refrigerant system. The $\eta_{ex}$ is highest for R1233zd(E), while it is lowest for R1234yf. With an increase in $T_{e}$, the total exergy destruction and the compressor power consumption decrease. However, the amount of input compressor power decreases more than the exergy destruction, resulting in a decrease in the $\eta_{ex}$. Furthermore, the exergy efficiency improvement $\eta_{ex,imp}$ is 2.60–12.72% for different refrigerants. Comparing various refrigerants shows that the maximum $\eta_{ex,imp}$ corresponds to R1233zd(E) + Al_2_O_3_ at $T_{e}$ of 20 °C, while the minimum $\eta_{ex,imp}$ is for R134a + Al_2_O_3_ at $T_{e}$ of −10 °C. 

#### 4.2.2. Condenser Temperature

**Figure 4:**
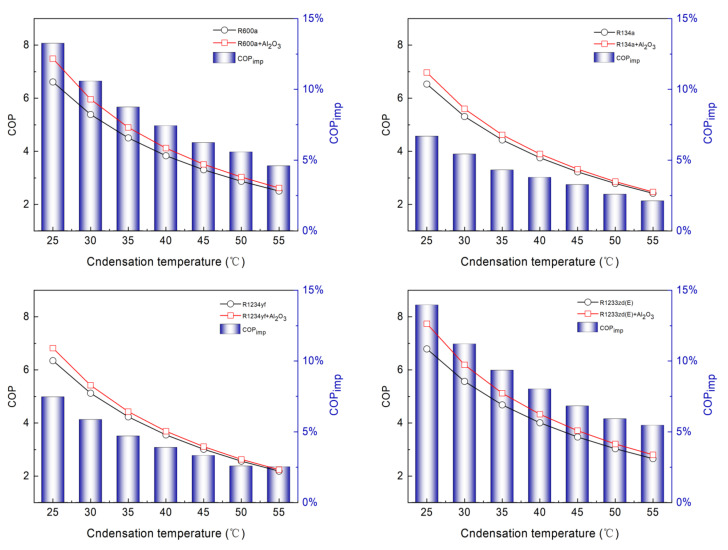
Variation of COP with condensation temperature at evaporation temperature of 0 °C.

Similarly, Figure 4 demonstrates the variation of COP with varying $T_{c}$ at $T_{e}$ of 0 °C. When the $T_{c}$ is 25 °C, the COP values of pure refrigerants R600a, R134a, R1234yf, and R1233zd(E) are 6.61, 6.53, 6.35, and 6.79, respectively. When the $T_{c}$ increases to 55 °C, the COP of pure refrigerants decreases to 2.51, 2.42, 2.19, and 2.66, respectively. The COP of pure refrigerants decreases by 0.137, 0.137, 0.139, and 0.138 for every 1 °C increase in $T_{c}$. For the nanorefrigerant system, the COP decreases significantly (from 7.49 to 2.62, 6.97 to 2.47, 6.82 to 2.24, and 7.74 to 2.80, respectively) when $T_{c}$ increases from 25 °C to 55 °C. When the $T_{c}$ increases by 1 °C, the COP decreases by about 0.162, 0.150, 0.153, and 0.165, respectively. The NR system is seen to be more sensitive to $T_{c}$ than the pure refrigerant system. The COP is highest for R1233zd(E), followed by those of R600a, R134a, and R1234yf. Compared with the pure refrigerants R600a, R134a, R1234yf and R1233zd(E), the ${\mathrm{COP}}_{\mathrm{imp}}$ for the NRs is increased by 4.59–13.26%, 2.11–6.69%, 2.52–7.47%, and 5.46–13.97%, respectively. This decrease is sharp for R1233zd(E) and lower for R134a. The lowest ${\mathrm{COP}}_{\mathrm{imp}}$ corresponds to R134a + Al_2_O_3_ at $T_{c}$ of 55 °C, whereas the highest ${\mathrm{COP}}_{\mathrm{imp}}$ is found with $T_{c}$ of 25 °C for R1233zd(E) + Al_2_O_3_. 

**Figure 5:**
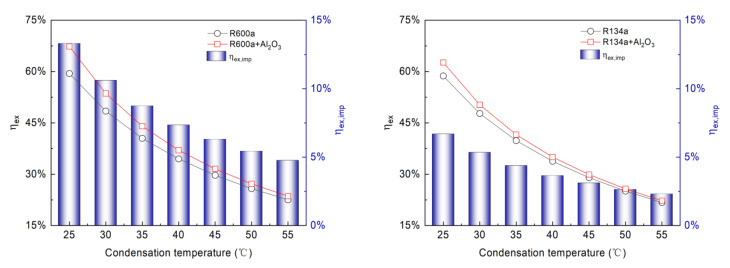

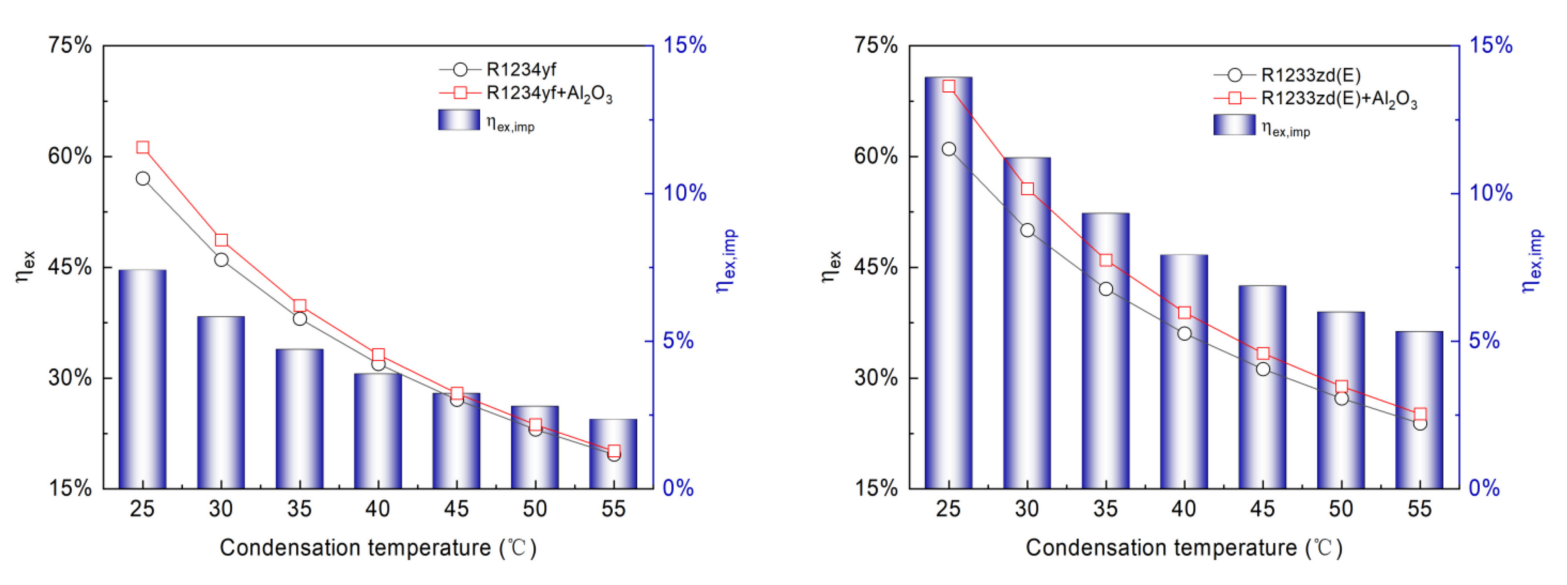
Variation of exergy efficiency with condensation temperature at evaporation temperature of 0 °C.

The $\eta_{ex}$ is shown for a range of $T_{c}$ from 25 °C to 55 °C in Figure 5. The $\eta_{ex}$ decreases from 59.43% to 22.51% for R600a, from 58.71% to 21.74% for R134a, from 57.04% to 19.64% for R1234yf, and from 61.04% to 23.86% for R1233zd(E), respectively. Similarly, for the NRs, when the $T_{c}$ is 25 °C, the $\eta_{ex}$ is 67.34%, 62.23%, 62.26%, and 68.54%, respectively; when the $T_{c}$ is increased to 55 °C, the $\eta_{ex}$ decreases to 23.58%, 22.24%, 20.01%, and 25.13%, respectively. This is mainly because the total exergy destruction and compressor power consumption increase with an increase in $T_{c}$, and the compressor power consumption is greater than the increased exergy destruction. A similar observation like the influence of evaporator temperature. It can be seen that $\eta_{ex}$ of the NR system can be increased by 4.75–13.31%, 2.30–6.68%, 2.34–7.40%, and 5.32–13.98%, respectively, compared to the pure refrigerant system. Comparing different refrigerants shows that the greatest improvement in $\eta_{ex}$ for R1233zd(E) occurs at $T_{c}$ of 25 °C, while the lowest $\eta_{ex,imp}$ corresponds to R134a at $T_{c}$ of 55 °C. 

#### 4.2.3. Nanoparticle Mass Fraction

**Figure 6:**
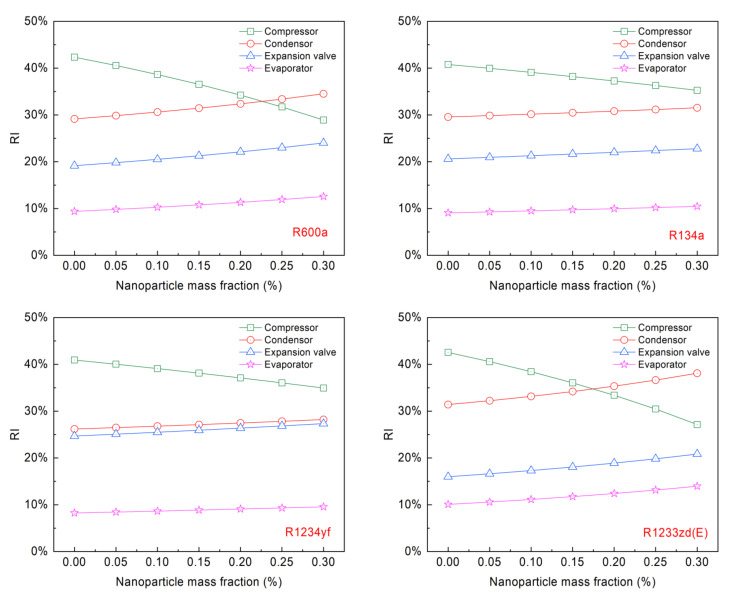
Nanoparticle mass fraction on relative irreversibility for cycle components.

The relative irreversibility (RI) of each component with different refrigerants is presented in Figure 6. The results show that the highest exergy destruction is the compressor, associated with 42.54% to 40.78% irreversibility with different pure refrigerants, while the lowest destruction is the evaporator, associated with 10.07% to 8.23% irreversibility. The reason for the compressor’s relatively higher RI value may be because of the highest compressor input power and isentropic losses. The compressor with a higher RI has greater potential to improve the system efficiency. With increasing the mass fraction of Al_2_O_3_ NPs, the exergy destruction of the compressor decreases, causing a descending trend in the compressor RI. When the NP increases to 0.20% wt.%, the compressor RI is less than condenser RI for the R1233zd(E) + Al_2_O_3_; and when the NP increases to 0.25% wt.%, the condenser RI is greater than compressor RI for R600a + Al_2_O_3_. 

**Figure 7:**
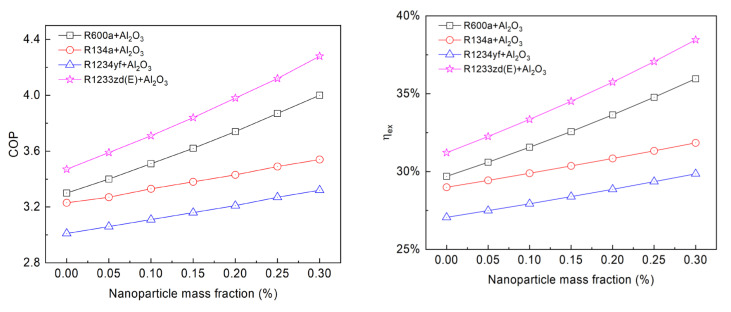
Variation of COP and exergy efficiency with nanoparticle mass fraction at condensation temperature of 45 °C and evaporation temperature of 0 °C.

Figure 7 shows the influence of the mass fraction of Al_2_O_3_ NPs on COP and $\eta_{ex}$ employing four different NRs at $T_{e}$ of 0 °C and $T_{c}$ of 45 °C. R1233zd(E) clearly shows the highest COP, followed by R600a, R134a, and R1234yf. The COP values of the pure refrigerants are 3.47, 3.30, 3.23, and 3.01, respectively; when the NP mass fraction increases to 0.30%, the COP values increase to 4.28, 4.0, 3.54, and 3.32, respectively. This is because more NPs per unit mass of refrigerant can increase the heat transfer capacity. R1233zd(E) + Al_2_O_3_ system has the largest COP enhancement of 23.24%, followed by the R600a + Al_2_O_3_ system with a COP enhancement of 21.07%, and followed by the R1234yf + Al_2_O_3_ system and R134a + Al_2_O_3_ system with COP enhancements of 10.30% and 9.77%, respectively. With a mass fraction of 0.30% Al_2_O_3_ NPs, the COP of R1233zd(E) + Al_2_O_3_ is 7.0% higher than that of R600a + Al_2_O_3_, it is 20.90% higher than that of R134a + Al_2_O_3_, and it is 28.92% higher than that of R1234yf + Al_2_O_3_. This trend remains the same as the mass fraction of Al_2_O_3_ NP varies. 

Moreover, the $\eta_{ex}$ increases linearly with the amount of Al_2_O_3_ NPs added. The maximum $\eta_{ex}$ is 38.46% for R1233zd(E) + 0.3% Al_2_O_3_, and the minimum $\eta_{ex}$ is 27.06% for pure R1234yf. The $\eta_{ex}$ for R134a and R1234yf increases steadily, while it increases rapidly for R1233zd(E) and R600a. With an increase in the mass fraction of Al_2_O_3_ NPs, the total exergy destruction and compressor power consumption decrease, but the reduced total exergy destruction is greater than the compressor power consumption, resulting in a rise in $\eta_{ex}$. The study shows that the R1233zd(E) with Al_2_O_3_ NPs may be used as a replacement for R134a because of zero ODP and low GWP. 

### 4.3. Optimization Analysis

**Figure 8:**
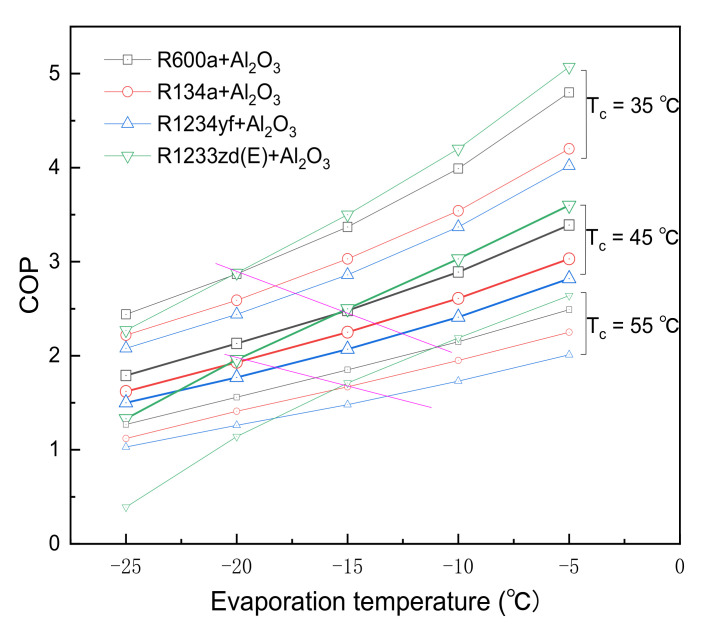
The COP variations of the nanorefrigerants with the evaporation temperature for three different condensation temperatures.

Figure 8 presents the COP variations with the evaporation temperature for three different condensing temperatures (the mass fraction of the NP is 0.3%). COP value increases more rapidly with increasing evaporation temperature for $T_{c}$ of 35 °C than the other condensation temperatures (45 °C and 55 °C). As the $T_{e}$ decreases and the $T_{c}$ increases, the COP of R600a + 0.3% Al_2_O_3_ may outperform R1233zd(E) + 0.3% Al_2_O_3_. Especially at $T_{e}$ of 20 °C and $T_{c}$ of 55 °C, the order of the COP is R600a + Al_2_O_3_, R134a + Al_2_O_3_, R1234yf + Al_2_O_3_, and R1233zd(E) + Al_2_O_3_, reaching 1.56, 1.41, 1.26, and 1.14, respectively. It could be said that R1233zd(E) is the worst alternative refrigerant for R134a under low-temperature refrigeration conditions.

## 5. Conclusions

Thermodynamic modeling using the energetic and exergetic analysis method is used to compare the effects of adding Al_2_O_3_ nanoparticles with different eco-friendly refrigerants (R600a, R134a, R1234yf, and R1233zd(E)) under different operation conditions. Therefore, the enthalpy of nanorefrigerants in the refrigeration cycle is calculated by the density prediction method in this study. Findings show that the thermal performance of a pure refrigerant system can be improved by adding nanoparticles. The main conclusions are obtained as follows:

(1) Compared with the pure refrigerants R600a, R134a, R1234yf, and R1233zd (E), the compressor work per unit mass with 0.1% Al_2_O_3_ nanoparticles is decreased by 2.96%, 2.80%, 2.89%, and 6.13%, respectively; and the corresponding COP is increased by 6.36%, 3.10%, 3.32%, and 6.92%, respectively. The performance of the nanorefrigerant cycle is enhanced by improving the heat transfer efficiency and lowering the compressor power consumption;
(2) The COPs and exergy efficiencies of R600a and R1233zd(E) are higher than that of R134a, while R1234yf is the lowest. R1233zd(E) + Al_2_O_3_ has the highest COP, followed by the R600a + Al_2_O_3_, R134a + Al_2_O_3_, and R1234yf + Al_2_O_3_ under the evaporation temperature ranges from −10 °C to 20 °C, the condensation temperature varies from 25 °C to 55 °C;
(3) Highest COP of 4.28 is obtained for R1233zd(E) + 0.3% Al_2_O_3_ at a condensation temperature of 45 °C and evaporation temperature of 0 °C. With a mass fraction of 0.3% Al_2_O_3_ nanoparticles, the COP of R1233zd(E) + Al_2_O_3_ is 7.0% higher than that of R600a + Al_2_O_3_, it is 20.90% higher than that of R134a + Al_2_O_3_, and it is 28.92% higher than that of R1234yf + Al_2_O_3_;
(4) The exergy efficiency increases linearly with the amount of Al_2_O_3_ nanoparticles added. The maximum exergy efficiency is 38.46% for R1233zd(E) + 0.3% Al_2_O_3_, and the minimum exergy efficiency is 27.06% for pure R1234yf;
(5) As the $T_{e}$ decreases and the $T_{c}$ increases, the COP of R600a + 0.3% Al_2_O_3_ may outperform R1233zd(E) + 0.3% Al_2_O_3_. Especially at $T_{e}$ of 20 °C and $T_{c}$ of 55 °C, the order of the COP is R600a + Al_2_O_3_, R134a + Al_2_O_3_, R1234yf + Al_2_O_3_, and R1233zd(E) + Al_2_O_3_, reaching 1.56, 1.41, 1.26, and 1.14, respectively.

The stability of nanofluids is, unfortunately, the greatest challenge for researchers in current technology. Namely, the nanorefrigerant system performance may decrease over time. Studies on equipment life cycle analysis with nanorefrigerants are necessary. More investigation is needed to investigate the application of nanoparticles in heat pumps. Additionally, researchers are advised to focus on hybrid nanoparticles (mixing two different nanoparticles or a composite nanoparticle into pure refrigerants). 

## Figures and Tables

**Table 1:** Overview of recent review articles on nanofluid study.

Authors	Remarks
Senthilkumar et al. (2020) [11]	Study the consequences of nanolubricants and nanorefrigerants and summarizes the methods to increase the heat transfer, enhancing the coefficient of performance and reduction in power consumption.
Yıldız et al. (2021) [12]	Summarize the preparation of nanofluids, the variation of thermophysical properties, the stability of nanofluids, impacts on the system performances of nanofluid usage, limitations, and challenges of nanoparticle usage, particularly in refrigeration systems.
Pinni et al. (2021) [13]	Study the thermal performance, improvement potentials, technical applications, and future challenges of various nanorefrigerants at different nanoparticle concentrations.
Praveen et al. (2021) [14]	An evolutionary timeline of nanorefrigerants and energy savings on compressor work by the addition of nanolubricants is discussed. Presents the dispersion techniques, stability, properties of boiling and condensation, migration phenomenon of nanoparticles, and various novel techniques to improve the performance of refrigeration systems.
Bilen et al. (2022) [15]	The effect of nanorefrigerants on the vapor compression refrigeration system performance is presented. The use of nanorefrigerants in vapor compression refrigeration systems improves the coefficient of performance up to 43.93% and 56.32% in the theoretical and experimental studies, respectively.

**Table 2:** Studies on the performance of nanorefrigerants in vapor-compression refrigeration systems.

Authors	Nanorefrigerant	Fraction	Evaluation
Bi et al. (2011) [19]	R600a with TiO_2_	0.1 & 0.5 $g\cdot L^{-1}$	The energy consumption of R600a + TiO_2_ is reduced by 9.6%.
Javadi et al. (2013) [20]	R134a with TiO_2_ and Al_2_O_3_	0.06 and 0.1 wt.%	Energy saving of 25% using 0.1% TiO_2_ nanoparticle.
Singh and Lal (2014) [21]	R134a with Al_2_O_3_	0.5 wt.%	The improvement in COP is maximum (7.2 to 8.5%). When Al_2_O_3_ nanoparticles are increased to 1 wt.%, COP has been found to be lower than that of pure R134a.
Mahbubul et al. (2015) [22]	R134a with Al_2_O_3_	5 vol.%	COP is 3.2% higher than pure R134a.
Mahdi et al. (2017) [23]	R134a with Al_2_O_3_	0.01 & 0.02 vol.%	The coefficient of heat transfer is enhanced by 0.54% and 1.1%, and COP is improved by 3.33% and 12%, respectively.
Sharif et al. (2017) [24]	R134a with SiO_2_	0–0.7 vol.%	Maximum and average COP enhancements are 24% and 10.5% with the addition of nanoparticles, respectively. The optimum volumetric fraction of nanoparticles is 0.05%.
Ande et al. (2018) [25]	R134a with CuO	1.6 wt.%	COP increased by 16.66%, and energy consumption decreased by 13.79%.
Dhamneya et al. (2018) [26]	R134a with TiO_2_	0.2 & 0.6 $g\cdot L^{-1}$	COP increased by 34.39% and 55.14%, respectively.
Alawi et al. (2019) [27]	R141b with Al_2_O_3_	1–4 vol.%	COP is increased by 15.13% in comparison to pure R141b.
Javadi et al. (2021) [28]	R134a with Al_2_O_3_	0.1 wt.%	The electricity consumption is 2.69% lower than that of the base fluid.

**Table 3:** Properties of selected refrigerants [39,40,41].

Refrigerant	Molecular Weight $\left(g\cdot mol^{-1}\right)$	Critical Temperature (°C)	Critical Pressure (MPa)	Boiling Point (°C)	Safety Group	ODP	GWP	Class
R600a	58.1	134.7	3.629	−11.75	A3	0	4	HC
R134a	102.0	101.1	4.06	−26.07	A1	0	1430	HFC
R1234yf	114.4	94.7	3.38	−29.49	A2L	0	4	HFO
R1233zd(E)	130.5	166.4	3.62	18.26	A1	<0.0004	4	HCFO

**Table 4:** Properties of the selected nanoparticles at a temperature of 25 °C [44].

Nanoparticle	Thermal Conductivity $\left(W\cdot m^{-1}\cdot {{}^{\circ}C}^{-1}\right)$	Density $\left(kg\cdot m^{-3}\right)$	Specific Heat $\left(kJ\cdot kg^{-1}\cdot {{}^{\circ}C}^{-1}\right)$	Particle Size (nm)	Average Price $\left(\$\cdot g^{-1}\right)$	Percentage Distribution
Cu	396.5	8958	0.239	50	0.60	3.16%
Al_2_O_3_	38.7	3970	0.765	48	0.69	36.84%
CuO	33.0	6315	0.530	77	0.41	12.63%
ZnO	27.2	5630	0.494	30–50	0.60	3.16%
TiO_2_	8.4	3900	0.692	28	0.94	16.84%
SiO_2_	1.38	2400	0.968	55–75	0.41	9.47%

**Table 5:** Correlations of thermophysical properties of nanorefrigerants.

Nanorefrigerants Property	Correlation	Reference
Density ($\rho_{NR}$)	$\rho_{NR}=\varphi \rho_{NP}+\left(1-\varphi \right)\rho_{R}\;\;\varphi =\frac{\omega \rho_{R}}{\omega \rho_{\mathrm{NP}}+\left(1-\omega \right)\rho_{R}}$	Bhattad (2018) [34]
Specific enthalpy ($h_{NR}$)	$h_{NR}=\omega h_{\mathrm{NP}}+\left(1-\omega \right)h_{R}$	Kosmadakis et al. (2019) [44]
Specific heat ($c_{p,\;NR}$)	$c_{p,\;NR}=\frac{\varphi \rho_{NP}c_{p,\;NP}+\left(1-\varphi \right)\rho_{R}c_{p,\;R}}{\rho_{NR}}$	Mahbubul et al. (2015 [22])
Dynamic viscosity ($\mu_{NR}$)	$\mu_{NR}=\mu_{R}\left(1+7.3\varphi +123\varphi^{2}\right)$	Maïga (2005) [45]
Thermal conductivity ($k_{NR}$)	$k_{NR}=k_{R}\frac{k_{NP}+\left(n-1\right)k_{R}-\left(n-1\right)\varphi \left(k_{R}-k_{NR}\right)}{k_{NP}+\left(n-1\right)k_{R}+\varphi \left(k_{R}-k_{NR}\right)}$	Hamilton and Crosser (1962) [46]

**Table 6:** Thermodynamic equations for the components of the refrigeration system [48].

Component	Thermodynamic Balance Equation
Compressor	$W_{comp}=\frac{m_{R}\left(h_{2,is,R}-h_{1,R}\right)}{\eta_{is}}-m_{NP}\left(h_{2,NP}-h_{1,NP}\right)\;\;\eta_{is}=0.65+0.015\frac{P_{2}}{P_{1}}-0.0015{\left(\frac{P_{2}}{P_{1}}\right)}^{2}$
Condenser	$Q_{con}=m_{R}\left(h_{2,R}-h_{3,R}\right)+m_{NP}\left(h_{2,NP}-h_{3,NP}\right)$
Expansion valve	$h_{3,NR}=h_{4,NR}$
Evaporator	$Q_{eva}=m_{R}\left(h_{1,R}-h_{4,R}\right)+m_{NP}\left(h_{1,NP}-h_{4,NP}\right)$

**Table 7:** The exergy destruction and exergy efficiency for the components of the refrigeration system [2,49].

Component	Exergy Destruction	Exergy Efficiency
Compressor	$Ex_{des,\;comp}=Ex_{1}-Ex_{2}+W_{comp}=m_{NR}T_{0}\left(s_{2,NR}-s_{1,NR}\right)$	$\eta_{ex,\;comp}=1-\frac{Ex_{des,\;comp}}{W_{comp}}$
Condenser	$Ex_{des,\;con}=\left(Ex_{2}-Ex_{3}\right)-Q_{c}\left(1-\frac{T_{0}}{T_{c}}\right)\;\;\;\;\;\;=m_{NR}\left[\frac{T_{0}}{T_{c}}\left(h_{2,NR}-h_{3,NR}\right)-T_{0}\left(s_{2,NR}-s_{3,NR}\right)\right]$	$\eta_{ex,\;con}=1-\left(\frac{Ex_{des,\;\;con}}{Ex_{2}-Ex_{3}}\right)$
Expansion valve	$Ex_{des,\;exp}=Ex_{3}-Ex_{4}=m_{NR}T_{0}\left(s_{4,NR}-s_{3,NR}\right)$	$\eta_{ex,\;exp}=\frac{Ex_{4}}{Ex_{3}}$
Evaporator	$Ex_{des,\;eva}=\left(Ex_{4}-Ex_{1}\right)+Q_{e}\left(1-\frac{T_{0}}{T_{e}}\right)\;\;\;\;\;\;=m_{NR}\left[\frac{T_{0}}{T_{e}}\left(h_{4,NR}-h_{1,NR}\right)-\left(s_{4,NR}-s_{1,NR}\right)\right]$	$\eta_{ex,\;eva}=1-\left(\frac{Ex_{des,\;\;eva}}{Ex_{4}-Ex_{1}}\right)$

**Table 8:** Validation of the model.

	$h_{1,NR}$ $\;(kJ\cdot kg^{-1})$		$h_{3,NR}=h_{4,NR}$ $\;(kJ\cdot kg^{-1})$		COP	
	Hussin et al. [50]	Model	Hussin et al. [50]	Model	Hussin et al. [50]	Model
R134a	394	394.37	258	259.40	3.24	3.20
R134a + 0.1%SiO_2_	393	394.48	249	259.21	3.43	3.49
R134a + 0.3%SiO_2_	395	394.52	250	258.83	3.72	3.66
R134a + 0.5%SiO_2_	395	394.55	243	258.45	4.00	3.85
Average deviation	0.06%		3.63%		−1.21%	

**Table 9:** Cycle parameters and calculated values.

Parameters	R600a-Al_2_O_3_		R134a-Al_2_O_3_		R1234yf-Al_2_O_3_		R1233zd(E)-Al_2_O_3_	
ω	0	0.1%	0	0.1%	0	0.1%	0	0.1%
$T_{1}$ (°C)	0	0.03	0	0.028	0	0.03	0	0.024
$T_{2}$ (°C)	52.22	50.04	61.63.	60.48	50.99	50.10	58.14	55.14
$T_{3}$ (°C)	45	44.67	45	44.82	45	44.83	45	44.67
$P_{1}$ (kPa)	157.96	159.12	292.80	293.11	315.82	316.15	48.11	48.16
$P_{2}$ (kPa)	604.45	599.39	1159.92	1154.46	1153.83	1148.90	252.14	249.62
$h_{1}$ ($\mathrm{kJ}\cdot {\mathrm{kg}}^{-1}$)	554.34	554.38	396.60	398.62	363.29	363.31	435.17	435.19
$h_{2}$ ($\mathrm{kJ}\cdot {\mathrm{kg}}^{-1}$)	628.58	624.48	440.36	439.20	396.84	395.89	478.07	475.45
$h_{3}=h_{4}$ ($\mathrm{kJ}\cdot {\mathrm{kg}}^{-1}$)	309.07	308.22	263.94	263.67	262.30	262.04	286.20	285.79
$\varphi_{1}$	0	0.0001	0	0.0004	0	0.0004	0	0.0001
$\varphi_{3}$	0	0.0132	0	0.0284	0	0.0255	0	0.0306
$\rho_{1}$ ($\mathrm{kg}\cdot m^{-3}$)	4.26	4.26	14.43	14.44	17.65	17.66	2.84	2.84
$\rho_{3}$ ($\mathrm{kg}\cdot m^{-3}$)	524.37	524.83	1125.05	1125.86	1012.65	1013.40	1212.77	1213.62
$q_{e}$ ($\mathrm{kJ}\cdot {\mathrm{kg}}^{-1}$)	245.26	246.16	134.66	134.95	100.99	101.27	148.97	149.39
$q_{v}$ ($\mathrm{kJ}\cdot m^{-3}$)	1044.08	1048.92	1942.91	1949.08	1782.17	1788.90	422.52	424.15
$w_{comp}$ ($\mathrm{kJ}\cdot {\mathrm{kg}}^{-1}$)	72.24	70.10	41.75	40.58	33.55	32.58	42.90	40.27
COP	3.30	3.51	3.23	3.33	3.01	3.11	3.47	3.71
$\eta_{ex}$	29.69%	31.56%	28.99%	29.89%	27.06%	27.93%	31.21%	33.35%

## Data Availability

Not applicable.

## Nomenclature

Symbols	
c	Specific heat, $\mathrm{kJ}\cdot {\mathrm{kg}}^{-1}\cdot K^{-1}$
E	Exergy rate, kW
h	Specific enthalpy, $\mathrm{kJ}\cdot {\mathrm{kg}}^{-1}$
k	Thermal conductivity, $W\cdot m^{-1}\cdot K^{-1}$
m	Mass flow rate, $\mathrm{kg}\cdot s^{-1}$
P	Pressure, kPa
Q	Thermal capacity, kW
s	Specific entropy, $\mathrm{kJ}\cdot {\mathrm{kg}}^{-1}\cdot K^{-1}$
T	temperature, K or °C
W	Power, kW
Greek	
η	Efficiency
μ	Dynamic viscosity, Pa ·s
ρ	Density, $\mathrm{kg}\cdot m^{-3}$
φ	Volume fraction
ω	Mass fraction
Abbreviations	
COP	Coefficient of performance
GWP	Global Warming Potential
HCs	Hydrocarbons
HCFOs	Hydrochlorofluoroolefins
HFCs	Hydrofluorocarbons
HFOs	Hydrofluoroolefins
NP	Nanoparticle
NR	Nanorefrigerant
ODP	Ozone Depletion Potential
RI	Relative irreversibility
VCRC	Vapor-compression refrigeration cycle
Subscripts	
1, 2, 3..	State point
con	Condenser
comp	Compressor
des	destruction
eva	Evaporator
exp	Expansion valve
imp	Improvement of energy efficiency
is	Isentropic process
NR	Nanorefrigerant
R	Refrigerant
